# The protective effect of pregnancy on risk for suicide attempt in a Swedish national cohort

**DOI:** 10.1038/s41598-026-49192-w

**Published:** 2026-04-22

**Authors:** Mallory Stephenson, Séverine Lannoy, Henrik Ohlsson, Jan Sundquist, Casey Crump, Kristina Sundquist, Alexis C. Edwards

**Affiliations:** 1 Department of Psychiatry, Virginia Institute for Psychiatric and Behavioral Genetics, Virginia Commonwealth University School of Medicine, Richmond, VA USA; 2 Center for Primary Health Care Research, Lund University, Malmö, Sweden; 3 University Clinic Primary Care, Skåne University Hospital, Lund, Region Skåne Sweden; 4 Departments of Family and Community Medicine and of Epidemiology, The University of Texas Health Science Center, Houston, TX USA

**Keywords:** Pregnancy, Suicide attempt, Mother, Father, Diseases, Health care, Medical research, Risk factors

## Abstract

We used national Swedish registry data to investigate the nature of the association between pregnancy and decreased risk for suicide attempt (SA). Analyses included pregnant individuals aged 18–35, biological fathers, and matched controls born 1975–1995. Data were available for between 366,125 and 2,129,244 females (depending on the model) and 359,622 males. We evaluated risk for SA as a function of pregnancy status using three methods designed to strengthen causal inference: a matched cohort design, co-relative analyses, and within-individual analyses. In within-individual analyses, we also examined potential moderators of the pregnancy-SA association, and we conducted separate analyses in the unborn children’s biological fathers. Across all analyses, pregnancy was associated with a lower risk of SA (ORs = 0.15–0.30). Moderation analyses indicated that the potentially protective effect of pregnancy was stronger in unmarried individuals, those with prior registration for externalizing or internalizing disorders, and younger individuals. Decreased SA risk was also observed during the postpartum period (ORs = 0.16–0.31) and in biological fathers (ORs = 0.67–0.82). These findings suggest that risk for non-fatal SA is substantially lower during pregnancy in expectant mothers and fathers, and the association between pregnancy and reduced SA risk may be causal.

**Supplementary Information:**

The online version contains supplementary material available at 10.1038/s41598-026-49192-w.

## Introduction

Pregnancy is a major life transition characterized by neurobiological changes^1^, physical changes, and the development of an emotional bond between the parents and unborn child (referred to as prenatal attachment)^2–4^. During pregnancy, the mother’s behavior has a direct impact on the fetus, which may serve as an intrinsic motivator for behavior change. Correspondingly, pregnancy has been associated with reductions in substance use and problems, lower caffeine consumption, and improved health behaviors^5–9^. Prior studies have also reported decreased suicidal behavior during pregnancy: The prevalence of suicide death is substantially lower in pregnant than in non-pregnant individuals^10–14^, and the prevalence of non-fatal self-harm during pregnancy is only 32 per 100,000 live births^15^ compared to around 215 per 100,000 women in the general population^16^. While many may intuit the association between pregnancy and decreased risk of suicidal behavior to be causal in nature, empirical evidence is lacking.

Pregnancy may have a causal protective effect on suicidal behavior through various factors such as maternal prenatal attachment^2^, the social role of motherhood^10,17^, hormonal changes during pregnancy, and/or concerns about the impact of a suicide attempt on the child’s safety and well-being^18^. Leading theories of suicide highlight social connectedness as a key protective factor for suicidal thoughts and behavior^19–22^; the intimate bond between pregnant individuals and fetuses might thus also contribute to a direct decrease in risk. Alternatively, the relationship between pregnancy and risk for suicidal behavior may be driven by confounding factors, such as physical health (e.g., individuals who exercise regularly are less likely to experience infertility and less likely to attempt suicide^23,24^), mental health (e.g., individuals with depression have higher risk of infertility and are more likely to attempt suicide^25,26^), or genetic confounders (e.g., shared genetic factors involved in physical health, mental health, and likelihood of pregnancy). Distinguishing between these possibilities will extend our understanding of the relationship between pregnancy and suicidal behavior.

In this study, we used data from Swedish population-based registries with national coverage to further investigate the nature of the association between pregnancy and risk for non-fatal suicide attempt (SA), which has been understudied relative to suicide death. We leveraged three complementary methods to strengthen causal inference from observational data: a matched cohort study, a co-relative design, and a within-individual approach. Further, we investigated whether sociodemographic characteristics, pre-pregnancy history of psychopathology, genetic liability for SA, and biological fathers’ SA status moderated the association between pregnancy and SA. We pursued these moderation analyses in an effort to identify any groups for whom pregnancy is *not* protective. We also examined moderation by birth year to offer insight into potential cohort effects. Finally, we investigated risk for SA among the unborn children’s biological fathers. Both mothers and fathers experience some degree of prenatal attachment, with inconsistent findings regarding parental differences in the level of attachment^27–29^. However, most prior studies of risk for suicidal behavior during the pregnancy period have focused only on mothers. Evaluating SA risk in both parents during the prenatal period may offer some insight into whether the potential protective effect of pregnancy is specific to mothers or reflects a broader effect of social connectedness and familial responsibility.

## Materials & method

This study relied on several Swedish population-based registers with national coverage. The Swedish registry includes data on the whole Swedish population and covers a wide range of areas such as population, health, and social factors. In this study, we relied on the medical birth register, which includes comprehensive information on pregnancy and birth-related outcomes in 98% of all births in Sweden^30^. The total population and multigenerational registers^31,32^ were used to identify demographic information during the pregnancy period (marital status, residential status, and education of the parents). Non-fatal SA and registrations for internalizing and externalizing disorders were retrieved from the Swedish patient register and primary care register. We also added information on alcohol and drug use disorders from the Swedish drug and criminal registers. Information from the different registers was linked using each person’s unique identification number. To preserve confidentiality, this identification number was replaced by a serial number. All methods were carried out in accordance with the ethical standards of the relevant regulations on human experimentation and with the Helsinki Declaration of 1975, as revised in 2008. We secured ethical approval for this study from the Regional Ethical Review Board of Lund University (IRB approval number 2008/471). Written informed consent for this study was deemed unnecessary as per the Swedish Ethical Review Authority guidelines because researchers accessed anonymized data from the registers. Therefore, informed consent was waived.

### Participants

We selected all females born in Sweden 1975–1995 who had at least one child registered in the Swedish Multigeneration Register, where the pregnant individual was likely first aware of being pregnant between the ages of 18 and 35. Since our follow-up period goes up to 2018, limiting our observation to females born in 1995 allowed us to include these individuals in our postpartum analysis with sufficient follow-up time, thus improving comparability of results between the different models. We assumed an average of 280 days from the end of the last menstrual period to birth, a 28-day menstrual cycle, and the strong suspicion of pregnancy arising 10 days after the missed menstrual period. Therefore, we estimated individuals were aware of being pregnant 242 days before birth (i.e., 280–[28 + 10]).

### Measures

Non-fatal SA was defined using International Classification of Diseases (ICD) codes referring to suicide and self-inflicted injuries (ICD-9), intentional self-harm (ICD-10), and events of undetermined intent, consistent with prior studies^33–36^.

Potential moderators of the association between pregnancy and SA included school grades at age 16, parental education, familial genetic risk scores for SA (FGRS_SA_), internalizing and externalizing disorders prior to the pregnancy period, age at pregnancy, marital status (individuals that got married during pregnancy or the pre-pregnancy control period were excluded), and SA in the father of the child during the pregnancy period. Additional details regarding the assessment of these variables are provided in Table S1. Briefly, FGRS_SA_ were calculated based on registrations for SA among 1st through 5th degree relatives. Scores accounted for the individual’s genetic relatedness to each relative (e.g., 0.5 for parents and full siblings, 0.125 for first cousins), the relative’s age at first registration for the outcome (when applicable), years of cohabitation, and differences in the number of relatives for whom information is available^37^ (Table S1). FGRS_SA_ have been associated with risk for SA in prior work^38^. Internalizing and externalizing disorders were coded as separate, binary variables: For internalizing disorders, individuals with any registration for major depression or anxiety disorders prior to pregnancy were coded as 1, and all others were coded as 0. For externalizing disorders, individuals with a registration for alcohol use disorder or drug use disorder were coded as 1, and all others were coded as 0.

### Statistical analysis

We used three complementary methods to study the association between pregnancy and SA: (i) a matched cohort design including related and non-related controls^39^, (ii) co-relative analyses^40^, and (iii) within-individual analyses^41^. All three approaches were implemented within a logistic regression framework. For (i), we first matched each pregnant female (hereafter referred to as “case”) to 5 non-pregnant control females with the same year and month of birth. The non-pregnant participants had to be alive and registered in Sweden at the time of the case’s pregnancy and not themselves registered as having a child within 9 months after the date of birth of the case’s child. The risk of SA was studied in both pregnant and non-pregnant individuals. For all non-pregnant participants, we studied risk for SA during the same period as the case. Then, we applied a similar matching approach with related individuals as non-pregnant controls, including female cousins, half-siblings, full siblings, and monozygotic (MZ) twins. To achieve a sufficient number of related non-pregnant participants, we allowed for up to three years of age difference between the pregnant case and the related non-pregnant control.

For (ii), we extended this approach by combining population, cousin, half-sibling, full-sibling, and MZ twin pairs discordant for pregnancy and performing co-relative analyses. The co-relative model, an extension of the co-twin control design^40,42^, evaluates whether differences between relatives in their pregnancy status predict differences in SA status. This method controls for all genetic and environmental factors shared by members of the relative pair, thereby strengthening causal inference. For example, if the association between pregnancy and SA remains statistically significant and similar in magnitude across relative pairs of varying genetic relatedness, this is consistent with a potential causal effect of pregnancy on SA risk. Conversely, attenuation of the effect size across relative pairs suggests that the association between pregnancy and SA may be partially or entirely driven by genetic and/or familial environmental factors (Fig. S1). We performed two co-relative analyses. In the first analysis, we allowed all coefficients for each relative type to be independent (i.e., observed model). In the second, we modeled the genetic resemblance assuming that it equaled: 0 for the population, + 0.125 for cousins, + 0.25 for half-siblings, + 0.5 for full siblings, and + 1 for MZ twins (i.e., predicted model)^43^. This predicted model allows for an improved estimation of the pregnancy-SA association across relative types. We compared the observed and predicted models using Akaike’s Information Criterion (AIC) and selected the one showing the best fit.

For (iii), we used a within-individual model comparing a pre-pregnancy control period (i.e., 242 days prior to the pregnancy) to the pregnancy period. We conducted sensitivity analyses with shorter and longer control periods (i.e., 6 months and 1 year prior to the pregnancy) to adjust for the fact that risk of SA may fluctuate across those periods (e.g., it can start decreasing when the individual plans on getting pregnant). We used conditional logistic regression, with a separate stratum for each proband (the pregnant person) during their own “case” and “control” periods. In other words, within each individual who was pregnant during the observation period, we compared their SA risk during a “control” (i.e., pre-pregnancy) period to their SA risk during the “case” (i.e., pregnancy) period. Importantly, because each individual serves as their own control, the within-individual design provides stringent control for potential confounders that vary between individuals or over extended periods of time within the same individual (e.g., factors related to fertility, including nutrition, physical exercise, substance use, and physical stress^44^). To extend our knowledge of the effect of pregnancy on SA risk, we also considered second and third pregnancies and examined rates of SA in the postpartum period. For postpartum analyses, our control period was the 242-day period prior to pregnancy, and we examined three 242 days postpartum periods: 0–242, 243–484, and 485–726 days after childbirth. We excluded pregnancies where the child died during the postpartum period.

In the context of the within-individual analyses, we further investigated potential moderators of the association between pregnancy and SA: school grades at age 16, parental education, FGRS_SA_, internalizing and externalizing disorders prior to the pregnancy period, age at pregnancy, marital status, and SA in the father of the child during the pregnancy period. In a supplementary analysis, we tested for potential cohort effects by evaluating whether the association between pregnancy and risk for SA was moderated by birth year. All moderation analyses were performed using an interaction term between the variable of interest and pregnancy status, with risk of SA as the outcome. Interactions were on the multiplicative scale^45^, consistent with the logistic regression framework.

Finally, we examined SA risk in biological fathers during the pregnancy period using a within-individual model. We compared rates of SA in fathers during the 242 days period prior to pregnancy to the pregnancy period. To investigate potential cohort effects, we tested whether the association between pregnancy and SA was moderated by the father’s year of birth.

These analyses were not pre-registered and should be considered exploratory. All statistical analyses were performed using SAS 9.4.

## Results

Information regarding the sample size for each analysis and the prevalence of SA is provided in Tables 1, 2, 3 and 4. Table S2 reports the descriptive statistics for the moderator variables used in within-individual analyses.

### Association between pregnancy and suicide attempt

Pregnancy was inversely associated with SA risk across all analysis types. In the matched cohort study (Table 1), we observed that the rates of SA were substantially lower in pregnant individuals when compared to both related and non-related controls.

**Table 1:** Matched cohort study of pregnancy and rates of suicide attempt (SA) in the population and groups of relatives.

	N		Prevalence of SA		OR (95% CI)
	Cases	Controls	Pregnancy	Control	
Matched cohort					
Population	354,874	1,774,370	0.05%	0.25%	0.19 (0.16; 0.22)
Matched relatives					
Cousins	131,535	156,490	0.05%	0.31%	0.17 (0.13; 0.22)
Half-siblings	7,609	8,396	0.13%	0.49%	0.26 (0.13; 0.52)
Siblings	48,523	50,569	0.04%	0.34%	0.11 (0.07; 0.18)
Monozygotic twins	782	782	0.13%	0.38%	0.33 (0.04; 3.21)

Pregnant individuals were matched to five unrelated control (i.e., non-pregnant) individuals based on month and year of birth. Pregnant individuals were also matched to related control individuals, including cousins, half-siblings, full siblings, and monozygotic twins.

*CI* confidence interval, *OR* odds ratio, *SA* suicide attempt.

**Fig. 1:**
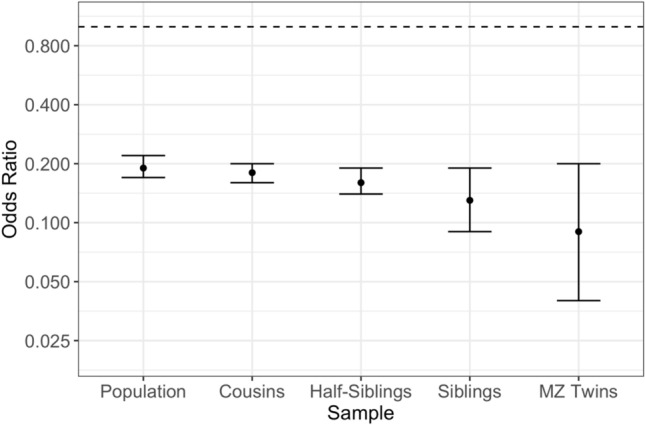
Co-relative analyses of the relationship between pregnancy and risk for suicide attempt. *Note*: Odds ratios are from the predicted model, as the predicted model provided better fit than the observed model (AIC for the predicted model = 1,614,865.8, AIC for the observed model = 1,614,867.9). The null hypothesis (odds ratio of 1) is shown as a dashed line. Error bars indicate 95% confidence intervals. MZ = monozygotic.

Results from the co-relative analyses are reported in Fig. 1. Pregnancy was significantly inversely associated with SA across all pairs of relatives—including MZ twins, the most conservative comparison—suggesting a potentially causal effect.

Within-individual analyses also indicated an inverse association between pregnancy and SA risk (Table 2): Rates of SA were lower during pregnancy relative to several pre-pregnancy control periods (ORs = 0.15–0.16). When considering the effects of second (OR = 0.30) and third (OR = 0.23) pregnancies, the association was slightly attenuated but remained statistically significant.

**Table 2:** Within-individual analyses of rates of suicide attempt (SA) in the pregnancy and postpartum periods.

	N	Prevalence of SA		OR
		Control period	Pregnancy period	95% CI
Pregnancy versus 242 days prior to pregnancy	366,445	0.33%	0.05%	0.16 (0.13; 0.18)
Pregnancy versus 6 months prior to pregnancy	366,445	0.32%	0.05%	0.15 (0.13; 0.18)
Pregnancy versus 12 months prior to pregnancy	366,445	0.33%	0.05%	0.15 (0.13; 0.17)
Second pregnancy versus 242 days prior to pregnancy	223,390	0.12%	0.04%	0.30 (0.23; 0.38)
Third pregnancy versus 242 days prior to pregnancy	50,183	0.22%	0.06%	0.23 (0.15; 0.35)

	N	Prevalence of SA		OR
		Control period	Postpartum period	95% CI
Analyses of postpartum periods				
0–242 days after childbirth versus 242 days prior to pregnancy	366,125	0.33%	0.05%	0.16 (0.14; 0.19)
243–484 days after childbirth versus 242 days prior to pregnancy	366,125	0.33%	0.08%	0.24 (0.21; 0.27)
485–726 days after childbirth versus 242 days prior to pregnancy	366,125	0.33%	0.10%	0.31 (0.28; 0.35)

CI = confidence interval; OR = odds ratio; SA = suicide attempt.

Finally, we explored whether sociodemographic and psychopathological factors previously associated with SA, including parental education, FGRS_SA_, marital status, pre-pregnancy history of externalizing and internalizing disorders, age at pregnancy, school grades, and SA in the unborn child’s biological father, moderated the association between pregnancy and SA risk (Table 3). The inverse association between pregnancy and SA was stronger in unmarried compared to married individuals, in those with prior registration for externalizing or internalizing disorders, and in younger individuals. A supplementary analysis indicated that the effect of pregnancy on SA risk was moderated by birth year, with stronger effect sizes in younger cohorts (Table S3).

**Table 3:** Potential moderators of the effect of pregnancy of risk for non-fatal suicide attempt in the context of within-individual analyses.

	Prevalence of SA		Interaction OR (95% CI) *p* value	OR (95% CI)
	Pregnancy period	Control period		
Parental education				
Low	0.06%	0.36%	1.05 (0.92; 1.19) *p* = .4724	0.15 (0.12; 0.18)
Mid	0.06%	0.31%		0.16 (0.14; 0.19)
High	0.02%	0.19%		0.18 (0.12; 0.26)
FGRS_SA_ (SD units)				
− 1.5 to − 0.5	0.02%	0.15%	1.02 (0.93; 1.13) *p* = .6650	0.15 (0.12; 0.19)
− 0.5 to 0.5	0.04%	0.25%		0.15 (0.12; 0.18)
0.5 to 1.5	0.08%	0.47%		0.15 (0.13; 0.18)
1.5 to 2.5	0.11%	0.61%		0.16 (0.13; 0.20)
2.5 to 3.5	0.21%	1.02%		0.16 (0.13; 0.19)
Married				
No	0.06%	0.35%	2.02 (1.22; 3.35) *p* = .0061	0.15 (0.13; 0.18)
Yes	0.03%	0.11%		0.30 (0.19; 0.49)
Externalizing behavior prior to pregnancy				
No	0.04%	0.18%	0.25 (0.17; 0.38) *p* < .0001	0.23 (0.19; 0.26)
Yes	0.31%	3.84%		0.06 (0.04; 0.08)
Internalizing behavior prior to pregnancy				
No	0.04%	0.16%	0.31 (0.22; 0.43) *p* < .0001	0.24 (0.21; 0.29)
Yes	0.17%	1.71%		0.07 (0.06; 0.10)
Age at pregnancy				
18	0.18%	0.74%	1.11 (1.07; 1.15) *p* < .0001	0.08 (0.06; 0.11)
19–25	0.08%	0.56%		0.18 (0.15; 0.20)
26–30	0.03%	0.14%		0.30 (0.24; 0.38)
31–35	0.04%	0.10%		0.51 (0.34; 0.76)
School grades (SD units)				
− 2.5 to − 1.5	0.13%	0.78%	1.14 (0.98; 1.33) *p* = .0877	0.11 (0.07; 0.20)
− 1.5 to − 0.5	0.08%	0.42%		0.13 (0.09; 0.19)
− 0.5 to 0.5	0.04%	0.19%		0.15 (0.11; 0.19)
0.5 to 1.5	0.02%	0.11%		0.17 (0.14; 0.20)
1.5 to 2.5	0.04%	0.07%		0.19 (0.16; 0.23)
Suicide attempt during pregnancy in fathers				
No	0.05%	0.29%	0.92 (0.45; 2.07) *p* = .8583	0.15 (0.13; 0.18)
Yes	1.35%	4.94%		0.14 (0.07; 0.31)

SA = Suicide Attempt, OR = Odds Ratio, 95% CI = 95% Confidence Interval, p = p-value. Interaction analyses were conducted on the multiplicative scale. Education, FGRS_SA_, age at pregnancy, and school grades were used as a continuous term in the models. These variables are binned for illustrative purposes only.

### Risk for suicide attempt in the postpartum period

The immediate postpartum period (0–242 days after childbirth) was associated with reduced risk of SA relative to the pre-pregnancy control period (OR = 0.16; Table 2). Effect sizes were attenuated during later postpartum periods (243–484 and 485–726 days after childbirth; ORs = 0.24 and 0.31, respectively) but remained significant.

### Association between pregnancy and suicide attempt in biological fathers

We explored whether pregnancy has a similar effect among biological fathers using a within-individual analysis (Table 4). Findings indicated that pregnancy was associated with a lower risk of SA in the father, but to a lesser extent than in the biological mother. In addition, the association between pregnancy and SA was stronger for biological fathers living in the same household as the pregnant individual (OR = 0.67 versus OR = 0.82 for fathers who lived in a different household).

**Table 4:** Within-individual analyses of rates of suicide attempt (SA) in fathers.

	N	Prevalence of SA		OR (95% CI)
		Pregnancy period	Control period	
Within-individual analyses				
Fathers not living with the pregnant individual	42,532	0.36%	0.43%	0.82 (0.70; 0.96)
Fathers living with the pregnant individual	317,087	0.09%	0.13%	0.67 (0.59; 0.74)
Analyses by year of birth				
< = 1975	125,780	0.09%	0.12%	0.75 (0.67; 0.84)
1976–1980	127,256	0.11%	0.12%	0.71 (0.65; 0.78)
1981–1985	78,469	0.15%	0.24%	0.68 (0.60; 0.76)
1986–1990	26,984	0.24%	0.36%	0.64 (0.54; 0.77)
1991–	2,677	0.30%	0.34%	0.61 (0.48; 0.78)

*CI* confidence interval, *OR *odds ratio, *SA* suicide attempt. There is a significant difference in the effect of pregnancy on suicide attempt risk in fathers who lived with the pregnant individual at the time of pregnancy versus did not live with the pregnant individual (interaction = 0.81, p = 0.03). There was not a significant effect of birth year (p = 0.182). Year of birth was included as a continuous term in the models but is binned for ease of presentation.

## Discussion

The present study used national registry data to investigate the magnitude and nature of the relationship between pregnancy and decreased risk for SA in both biological mothers and fathers. Below, we review six key takeaways from the results of this study.

First, consistent with previous studies of suicide attempt and death^10–15^, we observed an inverse association between pregnancy and rates of SA. The prevalence of SA was 50 per 100,000 in pregnant individuals, compared to 250 per 100,000 in population-based controls. Moreover, our findings were consistent with a potential causal association: Within monozygotic twin pairs discordant for pregnancy (i.e., in a model controlling for genetic and familial environmental confounders), the odds of SA were reduced by 91% in the pregnant twin. The magnitude of this association is substantial, particularly in view of the small effects typically observed in the suicide risk and protective factor literature^46^.

Second, the temporally discrete nature of pregnancy allowed us to conduct within-individual analyses, with each individual serving as their own control. In these analyses, rates of SA were consistently lower during pregnancy when compared to a range of pre-pregnancy time frames. Taken together, findings from the matched cohort design, co-relative analyses, and within-individual analyses provide converging evidence that the association between pregnancy and SA may be causal in nature, with the reduction in risk ranging from ~ 70 to 90% across analyses.

Third, we explored potential moderators of the association between pregnancy and risk for SA. We found that the inverse association between pregnancy and risk for SA was more pronounced for individuals who are not married, younger, and have a pre-pregnancy history of externalizing or internalizing behaviors. These findings suggest that pregnancy might be particularly protective for individuals who are otherwise at higher risk for suicidal behavior^47–50^; similar moderation effects have been observed in previous studies of pregnancy and risk of alcohol use disorder and drug abuse within the Swedish registries^5,8^. Notably, these results may partially reflect available resources for identifying and managing mental illness during pregnancy in Sweden. Swedish midwives are trained to screen for and identify symptoms of depression or anxiety, with extra attention given to women who are socially vulnerable, lack a strong social support network, or have a history of mental illness. Many maternity clinics have access to psychologists, and, if necessary, maternity clinics can collaborate with primary health care centers (for treatment of mild anxiety/depression) or psychiatric clinics (for more severe symptoms). These sources of support are offered free of charge for pregnant women and likely contribute to decreased SA risk.

Interestingly, however, there was no evidence to suggest that the effect of pregnancy varies according to genetic risk for SA. The effect size for the association between pregnancy and SA risk was quite stable, and 95% confidence intervals overlapped across levels of genetic liability. These results further support the consistent and pronounced impact of pregnancy on SA risk.

Fourth, we tested moderation of the association between pregnancy and SA by birth year to offer insight into potential cohort effects. In these analyses, risk for SA decreased more sharply during pregnancy in younger cohorts when compared to older cohorts. This observation may be driven by social changes related to childbearing and motherhood—for example, declines in the rate of unintended pregnancies, which are associated with higher rates of maternal depression, experiences of interpersonal violence, and substance use when compared to planned pregnancies^51–54^. Other, and non-mutually exclusive, explanations are that these findings may be attributable to cohort differences in the prevalence of SA, trends regarding medical care or resources available following an attempt, or changes in registry coverage over time.

Fifth, using within-individual analyses, we demonstrated that the reduction in SA risk persists into the postpartum period. Rates of SA during the immediate postpartum period (0–242 days after childbirth) were comparable to those observed during the pregnancy period, such that the odds of a non-fatal SA were reduced by 84% compared to the 242-day period prior to pregnancy. The magnitude of this association was somewhat attenuated during later postpartum periods but remained significant. These findings suggest that the protective effect of pregnancy does not merely reflect a time-limited desire to reduce fetal exposure to potentially harmful behaviors. Rather, the emotional bond that develops with a child across pregnancy and the transition to parenthood may yield broader and potentially long-term reductions in SA risk. Additional studies are needed, however, to further characterize SA risk across the transition to parenthood and to identify any subgroups for whom parenthood may not be protective.

Finally, we studied SA risk in the child’s biological father to offer further insight into the mechanism of the pregnancy-SA association. Using within-individual analyses, we demonstrated that biological fathers’ odds of SA are reduced during the pregnancy period, though to a lesser degree than observed for pregnant mothers. These findings suggest that a desire to reduce fetal exposure to harmful behaviors is not the only mechanism explaining the association between pregnancy and SA. The protective effect of pregnancy was also stronger in fathers who resided with the pregnant mother, supporting the hypothesis that expecting a child may foster a sense of social connectedness (e.g., with the biological mother and/or developing fetus). Nonetheless, the fact that there was some reduction of risk for biological fathers who did not reside with the pregnant individual suggests that the social role of parenthood and a sense of family responsibility may also protect against suicidal behavior, a possibility first raised by Emile Durkheim^55^.

These findings should be considered within the context of several limitations. First, our identification of SA is limited to those that require medical attention, and some attempts during pregnancy may have been missed. Another limitation of the registry data is the change in our ability to capture specific events over time. In this study, the prevalence of SA was 0.18% in individuals born 1975–1980 and 1.12% in individuals born 1991–1995. This may be partly explained by the fact that we have more registers available, more geographical areas contributing data, and, therefore, better coverage for younger cohorts. In addition, we do not precisely know when individuals realized they were pregnant and whether those pregnancies were planned. Taken together, the current estimates should be considered conservative. Second, data on prenatal attachment, relationship satisfaction, and broader social support are not available in the Swedish registries. It will be important for future studies to evaluate explanatory mechanisms for the strong and potentially causal relationship between pregnancy and SA risk. These efforts may enable the identification of specific mechanisms and pathways that can inform actionable prevention and intervention strategies in clinical care. Finally, our analyses were conducted in the Swedish population and may not be generalizable to other countries. The social resources and access to universal healthcare (including maternal healthcare that is free of cost) may contribute to stronger reductions in suicidal thoughts and behaviors during pregnancy in Sweden.

## Conclusion

While causal conclusions drawn from observational data are always tentative, this study offers converging evidence that pregnancy is causally associated with reduced SA risk. Inverse associations were strong, consistent across all analyses, and robust to potential confounders. The pregnancy-SA relationship may be partially explained by concerns about the impact of an attempt on the developing fetus’ safety and well-being. However, this protective effect extends beyond the pregnancy period and to biological fathers, suggesting that a broader sense of social connectedness and family responsibility also plays an important protective role.

## Supplementary Information

Supplementary Information.

## Data Availability

The Swedish registry data are not publicly available. However, we are willing to share the code related to the present project. Please contact Dr. Henrik Ohlsson (henrik.ohlsson@med.lu.se) for any questions related to the analyses.
