# Supplementary material for: The protective effect of pregnancy on risk for suicide attempt in a Swedish national cohort
Source: Sci Rep. 2026 Apr 22;16:18715. doi: 10.1038/s41598-026-49192-w (PMC13273045; doi:10.1038/s41598-026-49192-w)
Supplement: Supplementary file 1 — Supplementary Information. [file 41598_2026_49192_MOESM1_ESM.docx]

**SUPPLEMENTARY MATERIAL**

**The Protective Effect of Pregnancy on Risk for Suicide Attempt in a Swedish National Cohort**

Contacts:

[Mallory.Stephenson@vcuhealth.org](mailto:Mallory.Stephenson@vcuhealth.org)

[Severine.Lannoy@vcuhealth.org](mailto:Severine.Lannoy@vcuhealth.org)

**Table S1.** Definition of disorders and variables

| Variable/Disorder | Registers | Definition |
| --- | --- | --- |
| Suicide Attempt (SA) | National Patient Register and Primary Care Registry | IC10: X60-X84 and Y10-Y34  ICD9: E950-E959 and E980-E989 |
| Internalizing Disorder | The National Patient Register, Primary Care Registry | ICD-8: 296.2, 298.0, 300.4; ICD-9: 296.2, 296.4, 298.0, 300.4; ICD-10: F32, F33. (Major Depression)  ICD-8: 300.0, 300.2 ; ICD-9: 300A, 300C; ICD-10: F40, F41 (Anxiety Disorder) |
| Externalizing Behavior | The National Patient Register, Primary Care Registry; the Swedish Drug Register (2005-2018); the Swedish Mortality Register, and the Swedish Criminal Register (1973-2018) and the Swedish Suspicion Register (1998-2018) | Alcohol Use Disorder (AUD) was identified in the Swedish medical and mortality registries by ICD codes: ICD9: V79B, 305A, 357F, 571A-D, 425F, 535D, 291, 303, 980; ICD 10: E244, G312, G621, G721, I426, K292, K70, K852, K860, O354, T51, F10); in the Crime Register by codes 3005, 3201, which reflect crimes related to alcohol abuse; in the Suspicion Register by codes 0004, 0005 (Only those individuals with at least two alcohol-related crimes or suspicion of crimes from both Crime Register and Suspicion Register were included); in the Prescribed Drug Register by the drugs disulfiram (Anatomical Therapeutic Chemical (ATC) Classification System N07BB01), acamprosate (N07BB03), and naltrexone (N07BB04).  Drug abuse (DA) was identified in the Swedish medical and mortality registries by ICD codes (ICD8: Drug dependence (304); ICD9: Drug psychoses (292) and Drug dependence (304); ICD10: Mental and behavioral disorders due to psychoactive substance use (F10-F19), except those due to alcohol (F10) or tobacco (F17)); in the Suspicion Register by codes 3070, 5010, 5011, and 5012, that reflect crimes related to DA; and in the Crime Register by references to laws covering narcotics (law 1968:64, paragraph 1, point 6) and drug-related driving offences (law 1951:649, paragraph 4, subsection 2 and paragraph 4A, subsection 2). DA was identified in individuals (excluding those suffering from cancer) in the Prescribed Drug Register who had retrieved (in average) more than four defined daily doses a day for 12 months from either of Hypnotics and Sedatives (Anatomical Therapeutic Chemical (ATC) Classification System N05C and N05BA) or Opioids (ATC: N02A). |
| School Grades | The National School Registry. | Contains educational achievement (a grade point average) for all students at the end of basic high school (grade nine; usually at age 16). Students had an incentive to perform well in this school year because those with high grades were more likely to gain admission to the desirable upper secondary schools. For each year and by gender we standardized the grade score into a Z-score. From 1988 to 1997, the score was expressed on a scale between 1 (lowest) and 5 (overall mean was 3.2), and students were assessed by a peer referencing system. Grades awarded reflected the position of the student within Sweden and a set of correction factors were applied to ensure that the grades were equivalent between schools. Using this system, the grades had minimal grade inflation over time and were normally distributed. From 1998, the score was expressed on scale between 10 (lowest) and 320 (overall mean was 207) utilizing a criterion referenced system, in which students were assessed for their achievement of certain competencies. Scores were not standardized across schools or constructed to produce a normal distribution. |
| FGRS_SA_ | The National Patient Register, Primary Care Registry | The dataset for the calculations includes:  Column1 = Identification number of the proband (Born 1932-1995)  Column2 = Identification number of the relative (1st to 5th degree relatives)  Column3 = Proportion of shared additive genetic effects (0.03125 to 0.50) with the proband  Column4 = Year of Birth of relative  Column5 = Sex of relative  Column6 = Age at registration for trait  Column7 = Age at end of follow-up (2017-12-31 or age at death, or age at emigration whichever came first)  **Step 1**: Using all unique relatives with a registration for the disorder, we non-parametrically estimated the distribution of Age at first registration. The empirical distribution is used to obtain weights for relatives without a registration for the disorder, in order to account for the proportion of the time-at-risk period they had completed at the end of follow-up. For example, for relatives at age x at end of follow-up, the weight corresponds to the proportion of relatives registered for the trait that had been registration at age x. For relatives born prior to 1958 we subtracted age at the end of follow-up with the following formula: 1958 - Year of birth of relative. This modification was done in order to control for registration effects (i.e., most registers in Sweden start in 1973 suggesting that relatives from early birth cohorts do not have the possibility to be registered at younger ages). Note that all relatives with the disorder are weighted one.  **Step 2**: Transform the binary variable (trait yes/no) into a z-score based on the threshold for each trait. The underlying liability of the individual is not assessable. Instead, we estimated the mean of the underlying liability to obtain sex and birth decade specific Z-scores for relatives with the trait registration and relatives without the trait. We generate n random numbers from a N (0, 1) distribution and estimate the mean for relatives registered with the disorder (i.e., mean of the observations above the threshold) and for relatives without a registration (i.e., mean of all observation below the threshold). The thresholds are calculated for each decade of birth and sex.  **Step 3**: Correct for cohabitation effects. To estimate the cohabitation effect (i.e., “shared environment”), we created a database with all individuals in the Swedish population born in Sweden 1955-1990. We also included the number of years, during ages 0-15, that individuals resided in the same household as their biological father. We thereby were able to define two kinds of families: i) “not-lived-with” father families (offspring never resided for more than 1 year in the same household or in the same community as their biological father); ii) “lived-with” father (offspring resided a minimum of 13 year in the same household as their biological father. We performed a logistic regression model with the binary trait in offspring as outcome and the binary trait in father, type of father, and their interaction as predictors. We used the interaction term as the difference of effect between genes only and genes + environment. The same approach was performed for half-siblings where we compared those who were reared together versus reared apart  **Step 4**: Calculate the product for each relative using the four components:  I )Z-score (reflecting sex and year of birth adjusted rates)  ii) Weight (reflecting the proportion of risk period they had completed)  iii) Cohabitation effects  iv) Proportion of shared genetic effects (0.03125 – 0.5) with the proband  **Step 5**: Average the product calculated in step 4 across all relatives to a proband  **Step 6**: Correct for the number of relatives. We multiplied the results from step 5 with a shrinkage factor. Shrinkage factor (SF): B/(B+A/C). It produces more shrinkage if B and C are small and A is large.  (A) the variance of the z-score of the disorder across all relatives,  (B) the variance in the mean z-score across all probands,  (C) the weighted number of relatives for each proband (sum of Column 3 across each proband).  **Step 7:** Correct for difference by year of birth and county differences. There are 21 counties in Sweden. For each proband we used the county they had resided in during the maximum number of years (measured from 1969 and onwards) We standardized the risk score by year of birth and county of the proband into a z-score with mean 0 and SD 1. This was then used as the FGRS in the analyses |
| Married | Register of the total population | Date of registration of marriage |
| Same household as child | Register of the total population | Same FamilyID at year of birth of child |
| Parental Education | Multigenerational Register, LISA Register | Number of years of education are measured in 7 different levels  1 Pre-high school < 9 years  2 High School 9 years  3 Upper Secondary School < 3 years  4 Upper Secondary School 3 years  5 Post-secondary education < 3years  6 Post-secondary education 3 years or more  7 Research education (PhD). |

**Description of Registers**

*Multi-Generation Register*

The Multi-Generation Register is a register made up of persons who have been registered in Sweden at some time since 1961 and those who were born in 1932 or later. These are called index persons. The register contains connections between index persons and their biological parents. There are about 11 million index persons in the register. The Multi-Generation Register is a part of the register system for Total Population Register, where information comes from the National Tax Board. Every year, a new version of the register is created, including new index persons who immigrated or were born during the year. Information from the Multi-Generation Register may be disclosed for research and statistical purposes. For more information, see *Statistics Sweden, Background Facts, Population and Welfare Statistics 2017:2, Multi-generation register 2016. A description of contents and quality*. *Retrieved January 13, 2025 from https://www.scb.se/contentassets/95935956ea2b4fa9bcaab51afa259981/ov9999_2016a01_br_be96br1702eng.pdf*

*National Patient Register*

In the 1960's, the National Board of Health and Welfare started to collect information regarding in-patients at public hospitals, the National Patient Register (NPR). Initially it contained information about all patients treated in psychiatric care and approximately 16 percent of patients in somatic care. The register at that time covered six of the 26 county councils in Sweden. In 1984, the Ministry of Health and Welfare together with the Federation of County Councils decided a mandatory participation for all county councils. From 1987, NPR includes all in-patient care in Sweden. Since 2001, the register also covers outpatient doctor visits including day surgery and psychiatric care from both private and public caregivers. For more information, see *https://www.socialstyrelsen.se/en/statistics-and-data/registers/register-information/the-national-patient-register/*

*Primary Care Data*

We also used information from Primary Care. This is a research dataset including individual-level information on clinical diagnoses from primary health care centers. The figure below shows the percentage of the entire Swedish population that resides in counties with primary care data. In 2018 (the end of the follow-up period), the registers cover almost 100% of the population. For more information, see: Sundquist, J., Ohlsson, H., Sundquist, K., Kendler, KS. Common adult psychiatric disorders in Swedish primary care where most mental health patients are treated. BMC Psychiatry 17, 235 (2017). https://doi.org/10.1186/s12888-017-1381-4

*
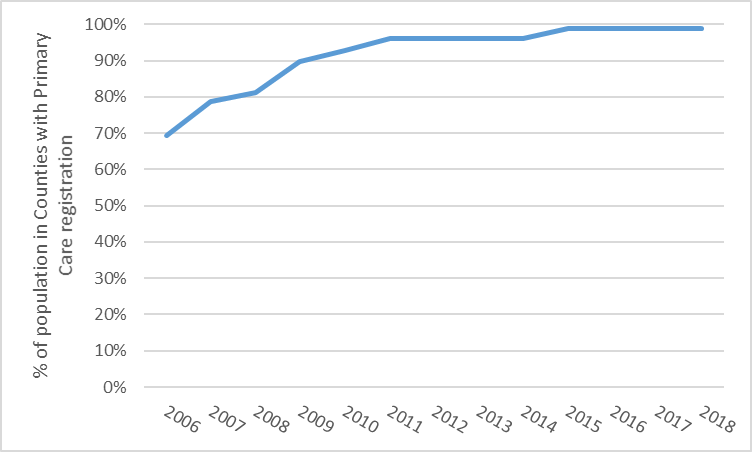
*

| **Continuous Variables** | | **M (SD)** |
| --- | --- | --- |
| Parental education | | 3.5 (1.3) |
| FGRS_SA_ (SD units) | | 0.0 (1.0) |
| Age at pregnancy | | 26.0 (4.0) |
| School grades | | -0.1 (1.0) |
| **Categorical Variables** | | **N (%)** |
| Married | |  |
|  | No | 280,922 (86.6%) |
|  | Yes | 43,632 (13.4%) |
| Externalizing behavior prior to pregnancy | |  |
|  | No | 354,254 (96.7%) |
|  | Yes | 12,191 (3.3%%) |
| Internalizing behavior prior to pregnancy | |  |
|  | No | 332,436 (90.7%) |
|  | Yes | 34,009 (9.3%) |
| Suicide attempt during pregnancy in fathers | |  |
|  | No | 360,559 (99.9%) |
|  | Yes | 445 (0.1%) |

**Table S2.** Descriptive statistics for moderator variables in within-individual analyses.

***Note.*** Within-individual analyses were conducted in pregnant individuals only (*N* = 366,445). Individuals who got married during the pregnancy period or pre-pregnancy control period were excluded.

**Table S3.** Moderating effect of birth year on the association between pregnancy and non-fatal suicide attempt in the context of within-individual analyses

|  | **N** | **Prevalence of SA** | | | **OR**  **(95% CI)** | |
| --- | --- | --- | --- | --- | --- | --- |
|  |  | Pregnancy period | | Control period |  |  |
| ***Analyses by year of birth (biological mother)*** | | | | | |  |
| 1975 – 80 | 199,092 | | 0.04% | 0.18% | 0.21 (0.16; 0.27) | |
| 1981 – 85 | 110,170 | | 0.05% | 0.32% | 0.17 (0.14; 0.19) | |
| 1986 – 90 | 50,249 | | 0.08% | 0.64% | 0.14 (0.11; 0.17) | |
| 1991 - 95 | 6,934 | | 0.25% | 1.12% | 0.11 (0.08; 0.16) | |

***Note***. CI = confidence interval; OR = odds ratio; SA = suicide attempt. Year of birth was included as a continuous term in the models but is binned for ease of presentation.

**Figure S1.** Interpreting patterns of associations in the co-relative model.


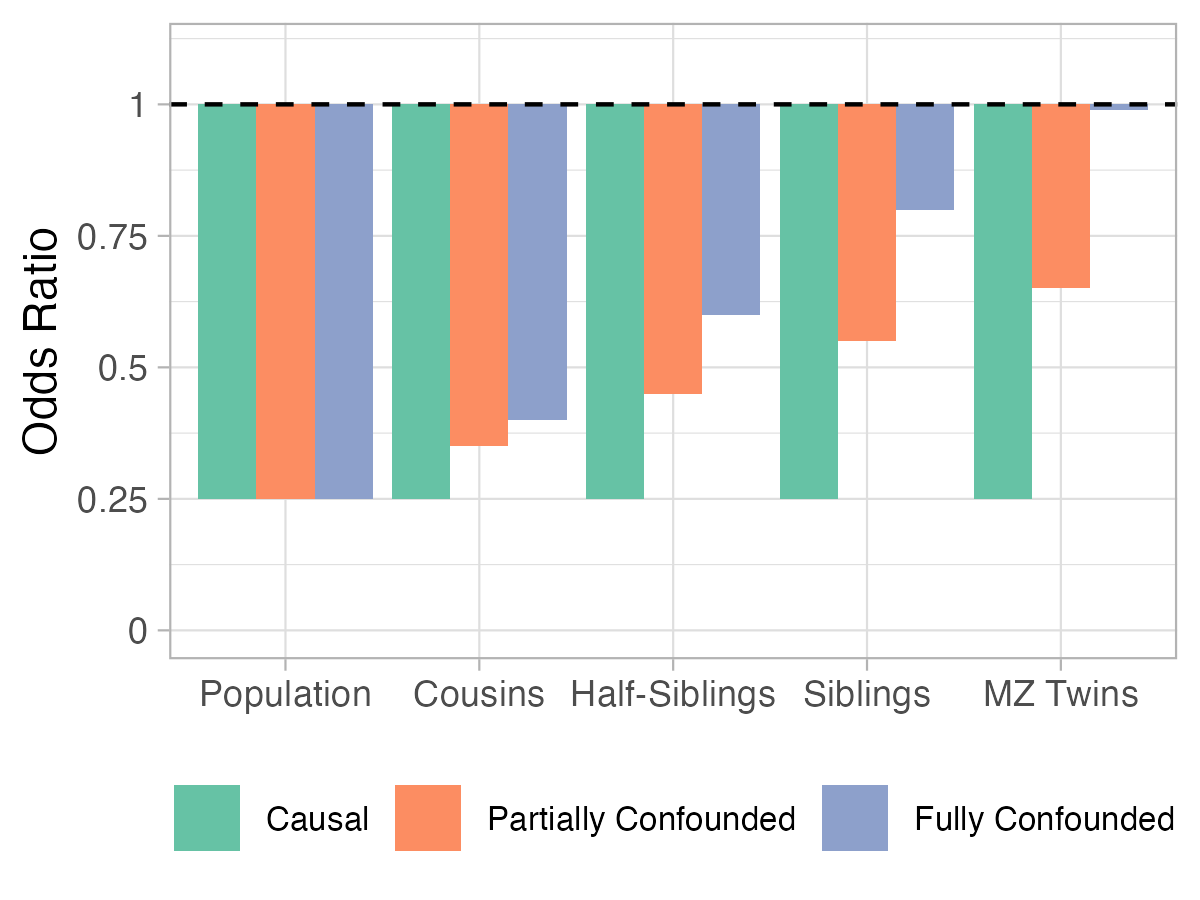


*Note.* This figure does not depict odds ratios from the current study; it is for illustrative purposes only. The co-relative model evaluates whether differences between relatives in their pregnancy status predict differences in their risk for suicide attempt (SA), thereby offering insight into the nature of the relationship between pregnancy and reduced SA risk. If pregnancy has a causal effect on SA, then the pregnancy-SA association should remain statistically significant and similar in magnitude across relative types. This pattern of results is represented by green bars. If the pregnancy-SA association is entirely driven by factors that are shared by family members, then the magnitude of the association should decrease across relative pairs of increasing genetic relatedness and be approximately equal to the null value (odds ratio of 1) within monozygotic twin pairs, who share a rearing environment and 100% of their genetic variation. The pattern of results consistent with a fully confounded association is shown as purple bars. Finally, an intermediate pattern of results can be observed, where the association between pregnancy and SA is partially attributable to familial factors and partially attributable to a causal effect. In this case, the magnitude of the association should decrease in relative pairs of increasing genetic relatedness but remain statistically significant within monozygotic twin pairs. This pattern of results is shown as orange bars. *Abbreviations.* MZ = monozygotic.
